# Short text classification approach to identify child sexual exploitation material

**DOI:** 10.1038/s41598-023-42902-8

**Published:** 2023-09-26

**Authors:** MHD Wesam Al-Nabki, Eduardo Fidalgo, Enrique Alegre, Rocio Alaiz-Rodriguez

**Affiliations:** 1 Department of Electrical, Systems and Automation Engineering, Universidad de León, León, Spain; 2 Researcher at INCIBE Spanish National Cybersecurity Institute, León, Spain

**Keywords:** Information technology, Computer science

## Abstract

Producing or sharing Child Sexual Exploitation Material (CSEM) is a severe crime that Law Enforcement Agencies (LEAs) fight daily. When the LEA seizes a computer from a potential producer or consumer of the CSEM, it analyzes the storage devices of the suspect looking for evidence. Manual inspection of CSEM is time-consuming given the limited time available for Spanish police to use a search warrant. Our approach to speeding up the identification of CSEM-related files is to analyze only the file names and their absolute paths rather than their content. The main challenge lies in handling short and sparse texts that are deliberately distorted by file owners using obfuscated words and user-defined naming patterns. We present two approaches to CSEM identification. The first employs two independent classifiers, one for the file name and the other for the file path, and their outputs are then combined. Conversely, the second approach uses only the file name classifier to iterate over an absolute path. Both operate at the character n-gram level, whereas novel binary and orthographic features are presented to enrich the text representation. We benchmarked six classification models based on machine learning and convolutional neural networks. The proposed classifier has an F1 score of 0.988, which can be a promising tool for LEAs.

## Introduction

The Council of the European Union (EU) has prioritized cybercrimes related to Child Sexual Abuse (CSA), considering them as the most severe crimes between 2020 and 2025^1^. According to The European Police Office, Child Sexual Exploitation Material (CSEM) is defined as sexual abuse of a person under 18 years old, producing images or videos of the abuse and distributing such content online^2^. Darknets, such as The Onion Router (Tor)^3^ and FreeNet^4^, Peer to Peer (P2P) networks, like eDonkey, and Instance messaging applications, Telegram as an example^5^, are environments where the interchange of CSEM seems to proliferate allowing pedophiles to share CSEM securely^6–8^. It is worth mentioning that during the COVID-19 outbreak, Interpol has reported a significant increase in exchanging CSEM in P2P and Darknet networks and online gaming and messaging applications^9^.

CSEM producers might save this content on their local computer machines, at least temporarily, or encrypted on a cloud server before sharing it with the consumers via Peer-to-peer (P2P) networks or live-streaming^10,11^. When the LEA inspects a home to analyze a suspect’s computer, a police officer will examine the files on the hard drive being inspected, trying to determine whether or not the suspected pedophile has stored CSEM on the computer. This task has to be accomplished accurately within a limited time.

A popular approach to identifying CSEM files is to use a file digital signature, which is known as a hash. Fortin et al.^12^ and Steel et al.^13^ explained the process of recognizing CSEM by comparing the hash of a new file with a database of hashes pre-calculates from known CSEM files. PhotoDNA^14^ is a software application developed by Microsoft to create hashes to known media files and can identify their instances even if they were modified slightly. It is robust to media-altering attacks like resizing, resizing, drawing, and watermarking. Nevertheless, the major drawback of hash-based techniques is the inability to detect new files without corresponding hashes in the database. Furthermore, the use of hashing techniques requires accessing the original files which are not available in our case, only their metadata, i.e. file names and paths. Machine learning algorithms are designed to overcome this shortage by learning CSEM naming patterns from the training set.

Among to potential techniques to recognize CSEM-related activity^12,15^, this work focuses on the file name information. In particular, it aims to build a File Classifier (FC) that decides whether a given file is CSEM related according to its name and absolute path only, without addressing the content as it is out of the scope of this paper. However, the main limitation of this approach is when the file name or path does not carry indicative information about the content or when the file name is encrypted, which prevents the FC from reaching the original name of the file. Nevertheless, the FC operates as a preliminary filter in a CSEM detection pipeline.

Building an automatic FC system is challenging for several reasons. First, a binary supervised algorithm requires training samples of Non-CSEM and CSEM files. However, there is no publicly available dataset for the latter class, and crawling child abuse material from P2P networks or Darknet is considered illegal in most countries. Therefore, CSEM file names must be legally obtained from LEAs. Second, a file name is typically a text of short length, which leads to a sparse representation of the samples owing to the massive number of features, whereas an instance is represented only with a few of them. Third, CSEM producers or consumers tend to invent a personalized file naming style, vocabulary, abbreviations, obfuscation, and acronyms to circumvent automatic detection tools. For example, given a file named “*!!!!yoB0yXX*”, the exclamation marks could refer to the age of a boy, and the letter *O* is replaced by the number zero. Hence, there is a high probability that this file is related to the abuse of a four years old boy. Finally, because file names do not have context, using state-of-the-art contextual embedding models becomes inefficient, such as BERT^16^, which showed superiority in text understanding tasks like sentiment analysis^17^.

**Figure 1:**
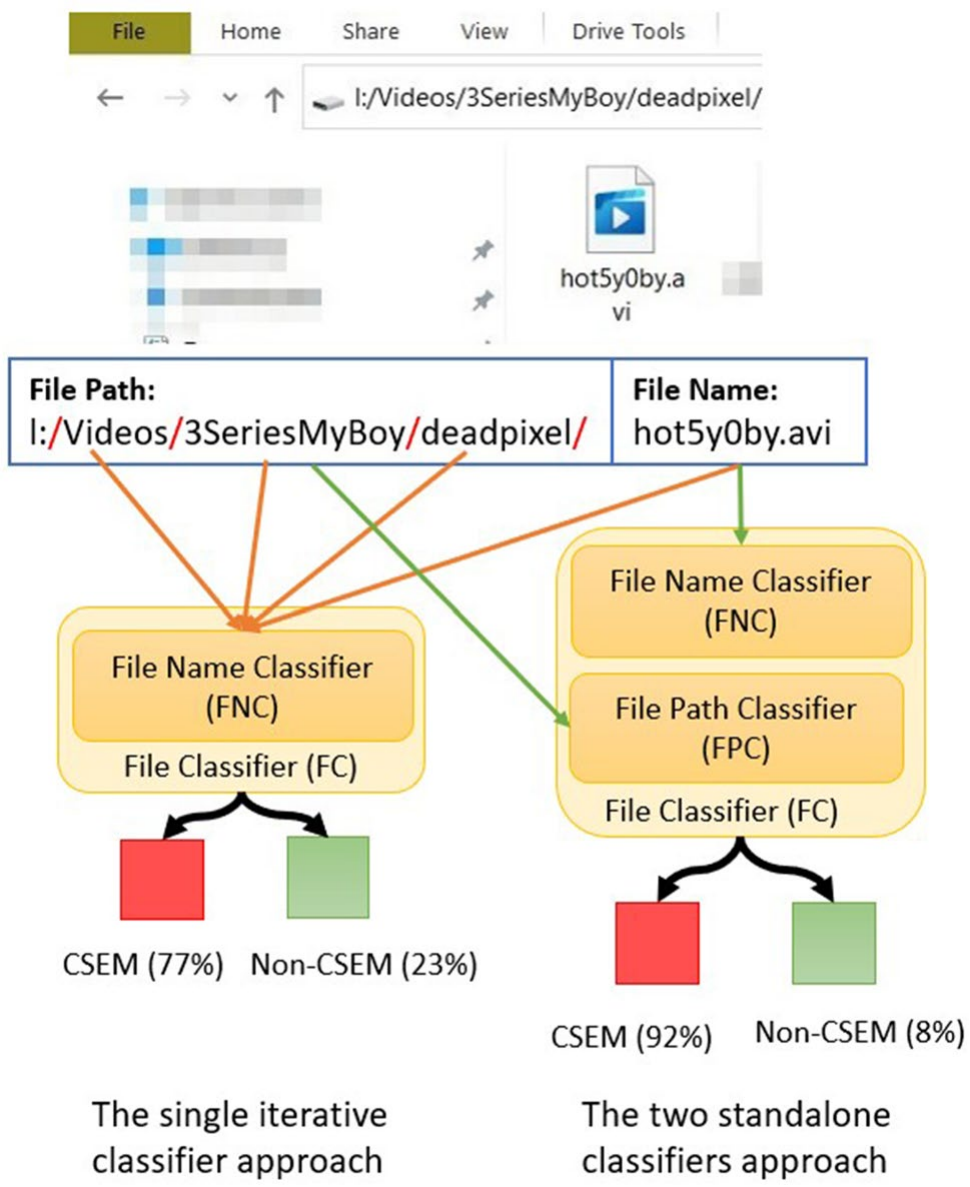
Two approaches to building the File Classifier system. The structure on the right shows how the two standalone classifiers approach works. In this configuration, FNC and FPC classify the file name and path, respectively. Then, their outputs are fused to determine the file category. The scheme on the left illustrates how the single-iterative classifier approach works. In this configuration, the FNC is used to iterate over the sub-directories of the absolute path.

Typically, an absolute path of a file consists of two components: the name and the absolute path. We propose two approaches for building the FC (Figure 1). The first uses a dedicated classifier for each component, that is, a File Path Classifier (FPC) to classify the absolute paths and a File Name Classifier (FNC) to classify file names, and their outputs are fused into a single score. The other approach uses the FNC to iterate over the sub-directories of the absolute path along the file name.

In the relationship between the path name and the file name, it is important to note that the path name is not derived from the file name or vice versa. The decision-making process for choosing path names is subjective and not directly linked to the specific names of the files contained within them. Moreover, it is worth considering that even if the same perpetrator or any individual were to encounter the same set of files, they would likely select different folder names based on various factors, such as the order in which the files were saved or the specific aspects, they wish to highlight in the folder name for easy retrieval later.

The main contributions of this paper are summarized as follows: We propose a framework for classifying files as CSEM or Non-CSEM based on the file name and path without addressing the content. We also extended the text of file names by appending two additional intermediate representations suitable for CSEM detection. The first is a novel binary representation that distinguishes character blocks from non-character blocks. The second is an orthographic feature that captures variations in the types of file name characters. To our knowledge, the orthographic feature has yet to be used before to code file names for text classification. Moreover, we built a dataset with 890K and 5.9M unique file names and paths, respectively, making it the most extensive dataset for classifying CSEM. Finally, we trained and tested the file classification model on a real-world dataset officially collected from the seized hard disks of CSEM producers and consumers. We also integrated our framework into a practical forensic tool used by a Spanish LEA under the 4NSEEK^18^ project to detect CSEM.

The remainder of this paper is organized as follows. Section "Related work" presents the related work. Section "Methodology" describes the proposed classification methodology. Section "Dataset construction" explains how the FNC and FPC datasets were created and their main features. In Section "Empirical evaluation", experiments are described. The results are discussed in Section "Discussion". Finally, Section "Conclusions and future work" presents the conclusions and our future research.

## Related work

Several researchers investigated the problem of CSEM detection using file names. Panchenko et al.^19^ attempted to normalize file names using Short Message Service (SMS) normalization techniques proposed by Beaufort et al.^20^. They trained a Support Vector Machine (SVM) classifier with normalized text and obtained an accuracy of $96.97\%$ on their dataset.

Peersman et al.^21^ proposed a framework called iCOP to detect CSEM in P2P networks. The first stage of their classification pipeline was a dictionary-based filter that was constructed manually and contained the CSEM keywords. They used a character n-gram of size two to four to capture more features of the file name, and a binary SVM as a classifier. Later, in their recent work^6^, they used a similar representation but benchmarked more classifiers such as SVM and Naive Bayes (NB). Owing to the lack of a public dataset for this task, they evaluated their proposal using a custom dataset. They observed that the SVM classifier could identify the CSEM file names with a recall rate of 0.43.

Al-Nabki et al.^22^ compared the use of machine learning classifiers, such as SVM and LR, that use character n-grams with Term Frequency-Inverse Document Frequency (TF-IDF) with deep learning classifiers that depend on convolutional neural networks (CNN). Specifically, they adopted two CNN models developed by Zhang et al.^23^ and Kim et al.^24^. The model of Zhang et al. was the best benchmarked CNN-based classifier and obtained an F1 score of 0.85, whereas the machine learning approach using the LR classifier obtained a slightly lower F1 score of 0.84. Pereira et al.^25^ compared several machine learning and deep learning models for classifying files using file names and paths. They conducted experiments on a 1, 010, 000 file path dataset from 55, 312 unique storage systems provided by Project VIC International. Similar to Al-Nabki et al.^22^, they found that the CNN character-based model proposed by Zhang et al.^23^ achieved the highest recall rate of 0.94.

The file path classification problem can be treated as a branch of URL classification because both share similar characteristics in terms of structure and the use of concatenated words. Several researchers have widely investigated this topic^26–29^. Sahingoz et al.^27^ used a URL classification approach to identify phishing websites by using URLs. They explored various features extracted manually from the URL to benchmark several machine-learning classifiers, such as SVM and Random Forest (RF).^29^ used a CNN by feeding it with word-level tokens extracted from the URLs.

## Methodology

This section introduces two approaches for designing the CSEM File Classifier (FC) and elaborates on each approach in detail.

### Two standalone classifiers approach

This approach has two classifiers: File Name Classifier (FNC) and File Path Classifier (FPC). Each classifier has its own dataset for training and testing.

#### File name classifier

The FNC attempts to enhance previous implementations explored in the literature by (1) enhancing the file name representation and (2) training on a larger dataset (Section "File name dataset"). Building an FNC involves three steps: text preprocessing, feature extraction, and classification.

The preprocessing attempts to extend the representation of a file name. We enriched the original file name using three techniques and concatenated their outputs to form an input to the next component in the classification pipeline. Table 1 shows the preprocessing and enrichment of the file name samples.

The text preprocessing function replaces special characters and numbers *#* and *$*, respectively, to reduce the sparsity of the features. For instance, a file named “*!!!!yoB0yXX*” will be transformed to “*####yoB$yXX*”. This technique is beneficial for detecting and eliminating duplicate samples that differ only in part of their names. For example, a folder in a seized computer could have more than 100 images named IMG01.png, IMG02.png, ... IMG100.png, whereas all these names repeat the same information as IMG##.png. However, we realized a significant portion of misclassified files do not have text indicating their category. For example, a sequence of random characters and digits such as “*DJ4MD9F34SE45EX8CH85YO*” and “*QBDD35HMF93DF5TVH4TD*”. Exploring the two and three grams of the former examples, we observe grams like “8YO”, “SE”, and “EX”, which might seem like indicators of suspiciousness. Nevertheless, looking at the full file name, the file name appears to be like a random sequence of characters.

This problem motivated us to enrich the representation of file names by adding a new binary representation that replaces each block of characters with one and zero for anything else, that is, digits and special characters. Hence, the above-mentioned examples are represented as “*1010101010101*” and “*101010101.jpg*”, while an example such as “*Hot3YoGirlOnBeach*” will be mapped to “*101*”. This representation can distinguish oscillations between characters and non-character blocks in the input text.

Additionally, we added an orthographic feature that maps capital letters, small letters, numbers, and special characters to unique tokens such as “C”, “c”, “N”, and “P”, respectively. As an example, a file name like “*!!!!yoB0yXX*” is mapped to “*PPPPccCNcCC*”.

**Table 1:** An example of preprocessing and enriching the representation of a file name.

Representation levels	Encoded text
Input text	!!!!yoB0yXX
Digit and special char.	####yoB$yXX
Binary representation	0101
Orthographic representation	PPPPccCNcCC
Output text	####yoB$yXX 0101PPPPccCNcCC

After extending the file name representation, we used the character n-gram to extract all the patterns of two to five consecutive characters of an input file name, which builds a set of tokens. We then applied the well-known TF-IDF technique^30^ because it gives higher weight scores to grams whose frequency is higher in a few file names and simultaneously decreases the weight of grams that frequently occur in many files. In this manner, it overcomes the issue of misspelled words or personalized naming styles in file names. Table 2 shows an example of two to five grams of a file name “*!!!!yoB0yXX*”. Furthermore, we set thresholds for the minimum and maximum term frequencies to eliminate the noisy tokens.

**Table 2:** An example of preprocessing and enriching a file name with two to five grams.

Original file name	!!!!yoB0yXX
Preprocessing	####yoB$yXX
2-grams	##, ##, ##, #y, yo, oB, B$, $y, yX, XX
3-grams	###, ###, ##y, #yo, yoB, oB$, B$y, $yX, yXX
4-grams	####, ###y, ##yo, #yoB, yoB$, oB$y, B$yX, $yXX
5-grams	####y, ###yo, ##yoB, #yoB$, yoB$y, oB$yX, B$yXX
Binary representation	0101
2-grams	01, 10, 01
3-grams	010, 101
4-grams	0101
5-grams	–
Orthographic representation	PPPPccCNcCC
2-grams	PP, PP, PP, Pc, cc, cC, CN, Nc, cC, CC
3-grams	PPP, PPP, PPc, Pcc, ccC, cCN, CNc, NcC, cCC
4-grams	PPPP, PPPc, PPcc, PccC, ccCN, cCNc, CNcC, NcCC
5-grams	PPPPc, PPPcc, PPccC, PccCN, ccCNc, cCNcC, CNcCC

Lastly, for the classification, we evaluated four machine learning classifiers: Logistic Regression (LR), Random Forest (RF), Naive Bayes (NB), and Gradient Boosted Trees (GBT), and two char-based CNN models proposed by Kim et al.^24^ and Zhang et al.^23^. We used these models to compare different implementations of the FNC classifier. Machine learning classifiers operate on previously extracted features, whereas CNN models automatically extract features from the text. In both cases, the FNC is a binary classifier that identifies whether the file name is CSEM.

#### File path classifier

The FPC is a supervised binary classifier that determines whether a given absolute path is CSEM. Like the FNC, the FPC consists of three components: file path preprocessing, feature extraction, and classification.

The preprocessing converts the input path into a string by replacing the slash sign (/) with space. Next, we replace special characters and digits with $\#$ and $\$$, respectively. Finally, capital letters are used to split the text. Table 3 illustrates the preprocessing procedure.

**Table 3:** Stages of the path preprocessing procedure.

Preprocessing stages	Text
Input text	/Home/Downloads/MyImages/MadridTrip_05_05_2020/IMG_1.png
Remove file name	l:/Videos/Voyeur/3SeriesMyBoy/deadpixel/
Replace (/) sign	l: Videos Voyeur 3SeriesMyBoy deadpixel
Replacing special char.	l# Videos Voyeur #SeriesMyBoy deadpixel
Split on capital letter	l# Videos Voyeur #Series My Boy deadpixel

We used the same feature extraction technique as the FNC, that is, n-grams at the character level, as described in Section "Two standalone classifiers approach". Finally, for the classification, we explored the six classification models, they are four machine learning classifiers: LR, NB, GBT, and RF, and two char-based CNN models.

#### Fusing file name and file path classifiers

This section introduces the aggregation of predictions of FNC and FPC classifiers into a single prediction value. The desired fusion strategy must be sensitive to potential CSEM in the file name or path. Hence, our fusion strategy returns the classifier result that has the highest CSEM confidence. For example, for a given sample *x*, the FNC predicts that it is CSEM with $20\%$ confidence and $80\%$ otherwise, while the FPC predicts that it is CSEM with $40\%$ confidence and Non-CSEM with $60\%$ confidence. In this case, the FPC confidence for CSEM is higher than the FNC one; therefore, the result of the FPC is the final output of the fusion. Formally, Eq. (1) explains the following procedure:

1 $$\begin{aligned} FC(x)=\left\{ \begin{array}{@{}ll@{}} FNC (x), &{} \text {if}\ FNC(x)_{CSEM}>FPC(x)_{CSEM} \\ FPC (x), &{} \text {otherwise} \end{array}\right. \end{aligned}$$

where *FC*(*x*) refers to the classification result of sample *x* and $FNC(x)_{CSEM}$ and $FPC(x)_{CSEM}$ refer to the classifier confidence regarding the CSEM class.

### Single-iterative classifier approach

Typically, the absolute path of a file is comprised of a sequence of folder names. This approach considers each folder as a standalone file name and uses the previously implemented FNC model for classification. Therefore, if an entry path has *N* sub-directories including the file name, the FNC is called *N* times, and *N* entries are classified. If any of these N entries were reported as CSEM, the complete path was considered CSEM. Otherwise, the entry is considered Non-CSEM. Unlike the majority voting approach, this technique is susceptible to any suspicious sub-directory name mentioned in the input path. The prediction complexity of this approach is proportional to the absolute path depth. Hence, for *M* samples, each of which has *N* sub-directories, the complexity is $O(N\times M)$.

## Dataset construction

The objective is to build a classifier to identify suspicious files via the name and absolute path without tackling the content. The dataset is a list of file names, their absolute paths, and their corresponding categories, which can be either CSEM or Non-CSEM.

It is worth mentioning that the path could be missing when the file resides on the root path of the hard drive, e.g. “/c00!_g1rl.mp4”. Moreover, files could have either representative names to indicate their category, such as “C:/users/cute_b0ys/ posing/4yoboy/h0o0ot.jpg” and “C:/users/Alex/Matlab.exe” or an indefinite name that does not carry information leading to the nature of the content, such as IMG_01.png, IMG_02.png, and 5T21764.jpg.

In addition, we observed that a file path could be shared among hundreds of file names when the files reside in one directory on the hard disk (Table 4). In addition, the folder may be empty, and hence a path name without file names. To obtain unique samples, we split the file names from their paths to obtain two lists: one for the unique file names and the other for the unique file paths.

**Table 4:** Examples of file paths dataset along with their corresponding file names.

File path	File name
l: Modeling\Silver Models\Sarah\Silver Starlets\Pink skirt 5\	pinkskirt-1-028.JPG
l:\Modeling\Silver Models\Sarah\Silver Starlets\Pink skirt 5\	pinkskirt-1-029.JPG
l:\Modeling\Silver Models\Sarah\Silver Starlets\Pink skirt 5\	pinkskirt-1-030.JPG
l:\Modeling\BD Modeling\Charming Models\	GX8E5675.jpg
l:\Modeling\BD Modeling\Charming Models\	GX8E5676.jpg

### File name dataset

For the Non-CSEM class, we used a dataset published by the National Software Reference Library (NSRL)^31^ which contains millions of file names. We randomly selected an initial subset of 800, 000 unique Non-CSEM examples, but after preprocessing and removing duplicate preprocessed samples (see the preprocessing procedure in 3.1), the number decreased to 519, 981.

Regarding the CSEM file names, one Spanish LEA gave us dumps of hard disks seized from criminals’ computers. The list contained 90, 000 CSEM samples. However, after preprocessing, the number of unique instances decreased to 37, 609. We split the preprocessed file names into 80/20 for the training and testing sets, respectively, as listed in Table 5.

### File path dataset

Similar to the file name classifier, the file path classifier has two classes: CSEM and Non-CSEM. For the Non-CSEM class, we gathered 3, 031, 802 unique paths from eight computer machines that host Non-CSEM files. After preprocessing, the number was reduced to 1, 141, 145. The same applies to CSEM file paths, where a Spanish LEA provided 2, 864, 105 file paths collected from six machines, after which the number was reduced to 924, 445 paths.

Unlike the file name samples, we did not split the training and testing sets on a fixed percentage, because the file path of the same machine shares a significant amount of text, such as the root drive name, username, and root folder name. We used the file paths of six Non-CSEM machines and four CSEM machines as a training set, and the remaining computer machines were left for testing, that is, the file paths of two Non-CSEM machines and two CSEM machines. Table 5 provides detailed information on the class size.

**Table 5:** Description of the used dataset to train the FNC and the FPC.

Classifier	Size	CSEM	Non-CSEM	Total
FNC	After preprocessing	37,609	519,981	557,590
	Training set size	30,129	408,708	438,837
	Testing set size	7,480	111,273	118,753
FPC	After preprocessing	924,445	1,141,145	2,065,590
	Training set size	31,925	80,928	112,853
	Testing set size	892,520	1,060,217	1,952,737

Finally, to test the performance of both the file name and file path models, we created a binary dataset of 50, 000 samples equally distributed between classes. We randomly sampled 50, 000 file paths and 50, 000 file names for each test set of paths and file names. We then created a balanced synthesized test set by fusing the two sets. A sample is considered CSEM if its name or path is sampled from a CSEM instance; otherwise, it is tagged non-CSEM.

## Empirical evaluation

### Experimental setting

The experiments were performed on a PC with an Intel(R) Core(TM) i7 processor with 32 GB of RAM and an Nvidia GeForce GTX 1060 GPU processor running on Windows 10. For ML classifiers, we used the Scikit-Learn toolkit, while for CNNs, we used an open-source solution that uses the Keras framework (https://github.com/chaitjo/character-level-cnn).

For the machine learning classifiers, we set the thresholds for the minimum and maximum gram proportions to 0.999 and 0.0005, respectively. Regarding the machine learning classifiers, we used the following settings empirically. For the LR classifier, we set the parameter *C* to 100, which refers to the inverse of regularization strength, and then activated the class weight parameter to consider the imbalance of the classes during training. For the GBT classifier, we used ten estimators similar to RF, setting the maximum depth to 3. The remaining parameters were left to their default values as set by the Scikit-Learn library. For the CNN models, we set the input dimension to 128 and activated early stopping after five iterations without improving the validation loss. To estimate the performance of the models, we report the performance of each classifier on a test set.

**Table 6:** Benchmark the performance of the FNC and the FPC using different classification models. Each model is evaluated in terms of Precision (P), Recall (R), and F1 score, and the values in bold refer to the best performance obtained.

Classifier	Class	File name classifier								File path classifier			
		Pure text				Orth and binary feat.				With preprocessing			
		P	R	F1	Support	P	R	F1	Support	P	R	F1	Support
RF	CSEM	1.000	0.183	0.309	7,510	0.996	0.381	0.551	7,480	0.788	0.702	0.742	1,060,217
	Non-CSEM	0.962	1.000	0.980	155,748	0.960	0.999	0.979	111,273	0.686	0.776	0.729	892,520
	Avg.	0.981	0.591	0.645	163,258	0.978	0.690	0.765	118,753	0.737	0.739	0.735	1952737
GRD	CSEM	0.901	0.027	0.054	7,510	0.983	0.867	0.921	7,480	0.929	0.940	0.935	1,060,217
	Non-CSEM	0.955	0.999	0.9770	155,748	0.991	0.999	0.995	111,273	0.928	0.915	0.922	892,520
	Avg.	0.928	0.5139	0.515	163,258	0.987	0.933	0.958	118,753	0.929	0.928	0.928	1,952,737
LR	CSEM	0.900	0.933	0.916	7,510	0.921	0.966	0.943	7,480	0.962	0.998	0.979	1,060,217
	Non-CSEM	0.996	0.995	0.995	155,748	0.997	0.994	0.996	111,273	0.997	0.953	0.974	892,520
	Avg.	0.948	0.964	**0.956**	163,258	0.959	0.980	**0.970**	118,753	0.979	0.975	0.977	1,952,737
NB	CSEM	0.752	0.898	0.818	7,510	0.881	0.904	0.892	7,480	0.997	0.997	0.997	1,060,217
	Non-CSEM	0.995	0.985	0.990	155,748	0.993	0.991	0.992	111,273	0.996	0.996	0.996	892,520
	Avg.	0.873	0.942	0.904	163,258	0.937	0.948	0.942	118,753	0.997	0.997	**0.997**	1,952,737
Zhang et al.	CSEM	1.000	1.000	1.000	150,448	1.000	1.000	1.000	116,632	0.958	0.988	0.973	1,060,217
	Non-CSEM	0.980	0.930	0.950	7,510	0.980	0.950	0.960	7,487	0.985	0.949	0.966	892,520
	Avg.	0.987	0.962	0.974	157,958	0.987	0.973	0.980	124,119	0.970	0.970	0.970	1,952,737
Kim et al.	CSEM	0.990	1.000	1.000	150,448	1.000	1.000	1.000	116,632	0.983	1.000	0.991	1,060,217
	Non-CSEM	0.990	0.920	0.950	7,510	0.990	0.950	0.970	7,487	1.000	0.980	0.989	892,520
	Avg.	0.992	0.960	**0.976**	157,958	0.992	0.975	**0.983**	124,119	0.991	0.990	**0.998**	1,952,737

### Empirical results

Table 6 reports the FNC and FPC performance with respect to the explored classifiers. Moreover, the table shows the impact of the binary and the orthographic feature used to enhance the file name representation. Our experiment found that Kim et al.’s model achieves the best performance for FNC and FPC classifiers with an F1 score of 0.983 and 0.998, respectively. Concerning the machine learning models, we found that the LR and the NB are the best models for the FNC and the FPC, with F1 scores of 0.970 and 0.997, respectively. It is worth mentioning that preprocessing the file name and extending it with binary and orthographic features boosted the F1 score of all models by $2\%$ at least.

While the improvement in the F1 score may appear modest in percentage terms, its real-world impact is significant. LEAs face strict time constraints in detecting this illicit material before they can seize the device. Even the slightest enhancement in the detection rate of any model could prove beneficial, as it increases the chances of LEAs achieving fast detection and enabling the seizure of hard drives containing CSEM. It is particularly valuable as it allows the detection of more files related to CSEM whose names do not clearly indicate their relationship to it. By using the folder name or the path name, additional files can be found that would otherwise go undetected. In some cases, where the perpetrator is careful with file names and even renames them to avoid revealing their contents but leaves some indication of their content in the folder, the file could be detected, leading to the apprehension of the criminal. The relevance goes beyond the small increase in the detection rate of the model, F1 score or precision, as it adds the possibility of detecting these files in the above-mentioned situations.

Given the superiority of Kim et al.’s model for the FNC and FPC as CNN-based models and the LR and NB as machine learning models, we used them to analyze the two fusion techniques described earlier. Table 7 shows that using the two standalone classifiers approach always surpasses the single-iterative classifier approach. Therefore, our results confirm the superiority of Kim et al.^24^ in identifying CSEM files, with an F1 score of 0.988. Nevertheless, the machine learning file classifier that uses LR for FNC and NB for FPC achieved a comparable F1 score of 0.984.

**Table 7:** A comparison between two fusion techniques: fusion 1 refers to the two standalone classifiers approach, and fusion 2 refers to the single-iterative classifier approach. We evaluate the best machine learning (ML) that uses LR for FNC and NB for FPC. For the CNN, we used Kim et al. architecture. The values in bold refer to the best prediction F1 score.

Model	Fusion	Category	Precision	Recall	F1	Support
CNN Model	Fusion 1	Non-CSEM	0.984	0.991	0.988	25,000
		CSEM	0.991	0.984	0.988	25,000
		Avg.	0.988	0.988	**0.988**	50,000
	Fusion 2	Non-CSEM	0.504	1.000	0.670	25,000
		CSEM	0.992	0.016	0.031	25,000
		Avg.	0.748	0.508	0.350	50,000
ML Model	Fusion 1	Non-CSEM	0.992	0.977	0.984	25,000
		CSEM	0.977	0.992	0.985	25,000
		Avg.	0.984	0.984	**0.984**	50,000
	Fusion 2	Non-CSEM	0.677	0.936	0.786	25,000
		CSEM	0.896	0.553	0.684	25,000
		Avg.	0.787	0.745	0.735	50,000

In addition, we evaluated model complexity in terms of the time required to predict 50, 000. We found that the two standalone classifier approach that used Kim et al.’s model for the FNC and FPC outperforms the other models with a prediction time of 15 and 156 seconds on a GPU and CPU machines, respectively, owing to the vectorization functionality. Hence, a typical machine with a GPU could classify 4, 167 files per second.

## Discussion

Among the experimented fusion techniques, we recognized the superiority of the two standalone classifiers approach which could be justified by the following. While reviewing the file path dataset, we noticed that some CSEM paths did not contain explicit CSEM-related words. In the first example in Table 4, there are terms that do not look CSEM related, such as “Sarah” and “Silver Starlets”. Still, their existence with other terms like “Starlets pink skirt”, and the number “5” could be an indicator of a photo session of a 5-years old girl called Sarah dressing in a pink skirt. Therefore, considering the full path and the name simultaneously allows the classifier to have a more comprehensive view of the content rather than focusing on the name of each sub-directory individually.

The high performance obtained by the classifier was surprising, particularly after using a large dataset compared with the literature review. This behavior can be attributed to two main reasons: the lack of variety in the CSEM file-naming patterns in the dataset. The dataset was obtained from one LEA, and the provided samples could be from a group of CSEM producers who use a similar naming convention. Another reason is the clear distinction between the vocabularies of both classes. For example, it is rare to find CSEM-related keywords, like “pedo” or “hot-baby”, in a dataset of collected from public sources, i.e. the Non-CSEM class. To address these limitations, we plan to request more than one LEA to provide us with their own dataset. However, this approach is difficult given the sensitivity of the operational data. Therefore, we plan to use a Federated Learning (FL) architecture^32^ which allows distributed training between different datasets without exchanging the dataset itself. Furthermore, we plan to include Non-CSEM examples that contain sex-related words, such as movie names, collected from torrent and pornography websites.

## Conclusions and future work

This paper presented an approach to identify files that could be related to Child Sexual Exploitation Material (CSEM) by analyzing their names and absolute paths. We proposed two approaches 1) building two standalone classifiers, a File Name Classifier (FNC) and a File Path Classifier (FPC), and then fusing their outputs into a single decision, and 2) dividing the absolute file path into a list of folder names and using the FNC to classify each. Our results strengthen the superiority of the former approach as it obtained an F1 score of 0.988. To build the FNC and FPC, we evaluated six classifiers, four using machine learning and two CNN models. For the FNC, we preprocessed the text and extended it with two new features: binary and orthography, which increased the F1 score by $2\%$, at least for all explored classifiers. Our experiments showed that the CNN model that operates at the character level could obtain F1 scores of 0.983 and 0.998 for FNC and FPC, respectively. This model can classify 50K files in 15 seconds on a typical GPU computer. The empirical evaluation was conducted using a dataset extracted from the file names and paths.

In future work, we look forward to enlarging the dataset by exposing the model to broader CSEM naming patterns from datasets of different law enforcement agencies. However, given the dataset sensitivity, we plan to use a Federated Learning architecture^32^. The assessment of Transformers, such as BERT^33^, on noisy and short texts will be evaluated shortly. Finally, text de-obfuscation is part of our immediate future research^34^.

## Data Availability

The dataset used during the current study has two classes, CSEM-related and Non-CSEM. The latter class is available from the corresponding author upon reasonable request. However, the CSEM-related file names contain sensitive data that cannot be shared.
