# Author Correction: Short text classification approach to identify child sexual exploitation material

**DOI:** 10.1038/s41598-023-45265-2

**Published:** 2023-10-19

**Authors:** MHD Wesam Al-Nabki, Eduardo Fidalgo, Enrique Alegre, Rocio Alaiz-Rodriguez

**Affiliations:** 1 Department of Electrical, Systems and Automation Engineering, Universidad de León, León, Spain; 2 Researcher at INCIBE Spanish National Cybersecurity Institute, León, Spain

Correction to: *Scientific Reports* 10.1038/s41598-023-42902-8, published online 26 September 2023

The Acknowledgements section in the original version of this Article was incorrect. It now reads:

“This research has been funded with support from the European Union’s Horizon 2020 Research and Innovation Framework Programme, H2020 SU-FCT-2019 under the GRACE project with Grant Agreement 883341. This publication reflects the views only of the authors, and the European Union’s Horizon 2020 Research and Innovation Framework Programme, H2020 SU-FCT-2019 cannot be held responsible for any use which may be made of the information contained therein.”

The original Article has been corrected.

